# Supplementary material for: Therapeutic targeting of fibrin–microglia interactions ameliorates Alzheimer’s disease-related hyperexcitability and brain network dysfunction
Source: bioRxiv. 2026 May 6:2026.05.01.722324. Preprint. [Version 1] doi: 10.64898/2026.05.01.722324 (PMC13174307; doi:10.64898/2026.05.01.722324)
Supplement: 1 [file NIHPP2026.05.01.722324v1-supplement-1.pdf]

bioRxiv preprint doi: https://doi.org/10.64898/2026.05.01.722324; this version posted May 6, 2026. The copyright holder for this preprint (which was not certified by peer review) is the author/funder, who has granted bioRxiv a license to display the preprint in perpetuity. It is made available under aCC-BY 4.0 International license.

SUPPLEMENTAL FIGURES

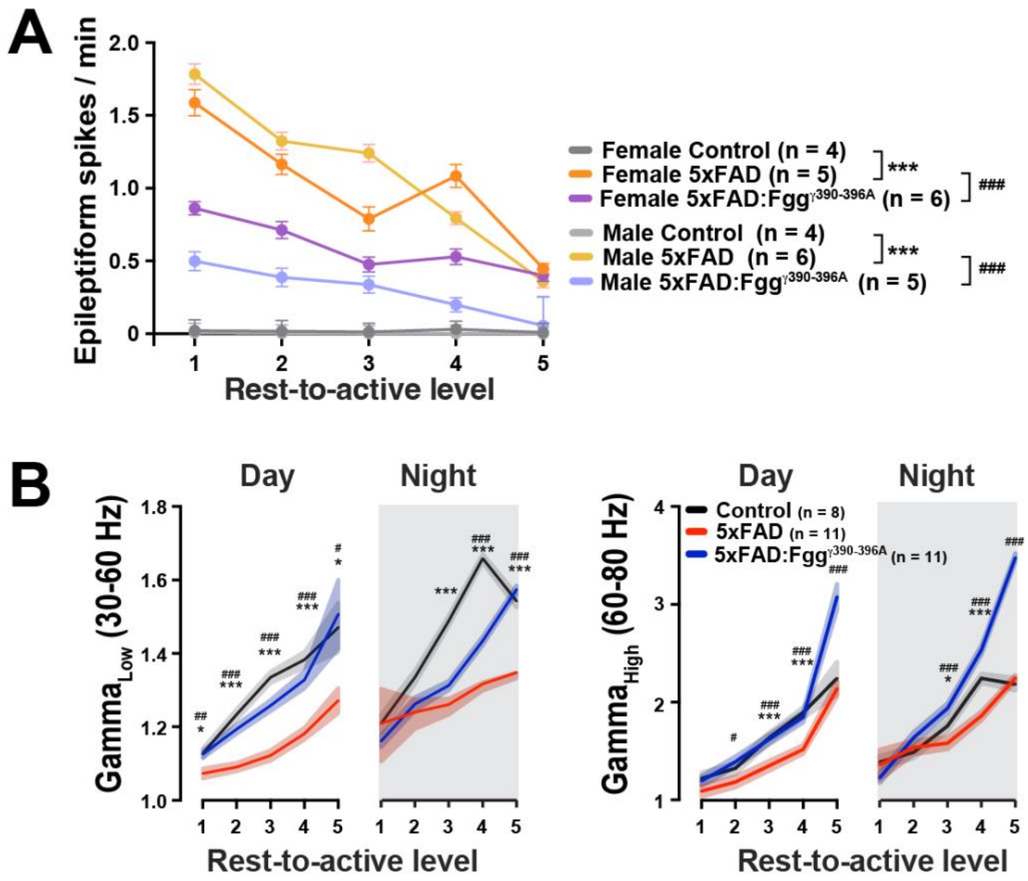

Figure S1. Related to Figure 2. 5xFAD-dependent alterations in both low and high gamma brain oscillations are ameliorated by blocking fibrin–microglia interactions in 5xFAD:*Fgg*$^{\gamma390–396A}$ mice. 10–13-month-old sex-mixed control (n = 8), 5xFAD (n = 11), and 5xFAD:*Fgg*$^{\gamma390–396A}$ (n = 11) mice were implanted with a wireless EEG/EMG transmitter to continuously monitor cortical brain activity for 7 days. (**A**) Epileptiform spikes by locomotor activity level (rest-to-active) and sex. Groups included: Control (n = 8; 4 females, 4 males), 5xFAD (n = 11; 5 females, 6 males), and 5xFAD:*Fgg*$^{\gamma390–396A}$ (n = 11; 6 females, 4 males) mice. Epileptic spikes were strongly modulated by locomotion in 5xFAD and 5xFAD:*Fgg*$^{\gamma390–396A}$ mice, with periods of low locomotor activity having higher epileptic spike rates and vice versa. Relative to 5xFAD mice, 5xFAD:*Fgg*$^{\gamma390–396A}$ mice had reduced epileptic activity.

(**B**) Low gamma (30-60 Hz) and high gamma (60-80 Hz) power are both reduced during the day and night in 5xFAD mice compared to controls, but rescued in the 5xFAD:*Fgg*$^{\gamma390–396A}$ mice. Values are mean ± SEM; P values by Generalized Linear Mixed Model (GLMM) accounting for repeated measures, individual

bioRxiv preprint doi: https://doi.org/10.64898/2026.05.01.722324; this version posted May 6, 2026. The copyright holder for this preprint (which was not certified by peer review) is the author/funder, who has granted bioRxiv a license to display the preprint in perpetuity. It is made available under aCC-BY 4.0 International license.

differences (random factor), fixed factors (day/night and activity levels), and Bonferroni post hoc test for multiple comparisons. $^{*}p < 0.05$, $^{**}p < 0.01$, and $^{***}p < 0.001$ for Controls vs. 5xFAD; $^{\#}p < 0.05$, $^{\#\#}p < 0.01$, and $^{\#\#\#}p < 0.001$ for 5xFAD vs. 5xFAD:*Fgg*$^{\gamma390–396A}$.

bioRxiv preprint doi: https://doi.org/10.64898/2026.05.01.722324; this version posted May 6, 2026. The copyright holder for this preprint (which was not certified by peer review) is the author/funder, who has granted bioRxiv a license to display the preprint in perpetuity. It is made available under aCC-BY 4.0 International license.

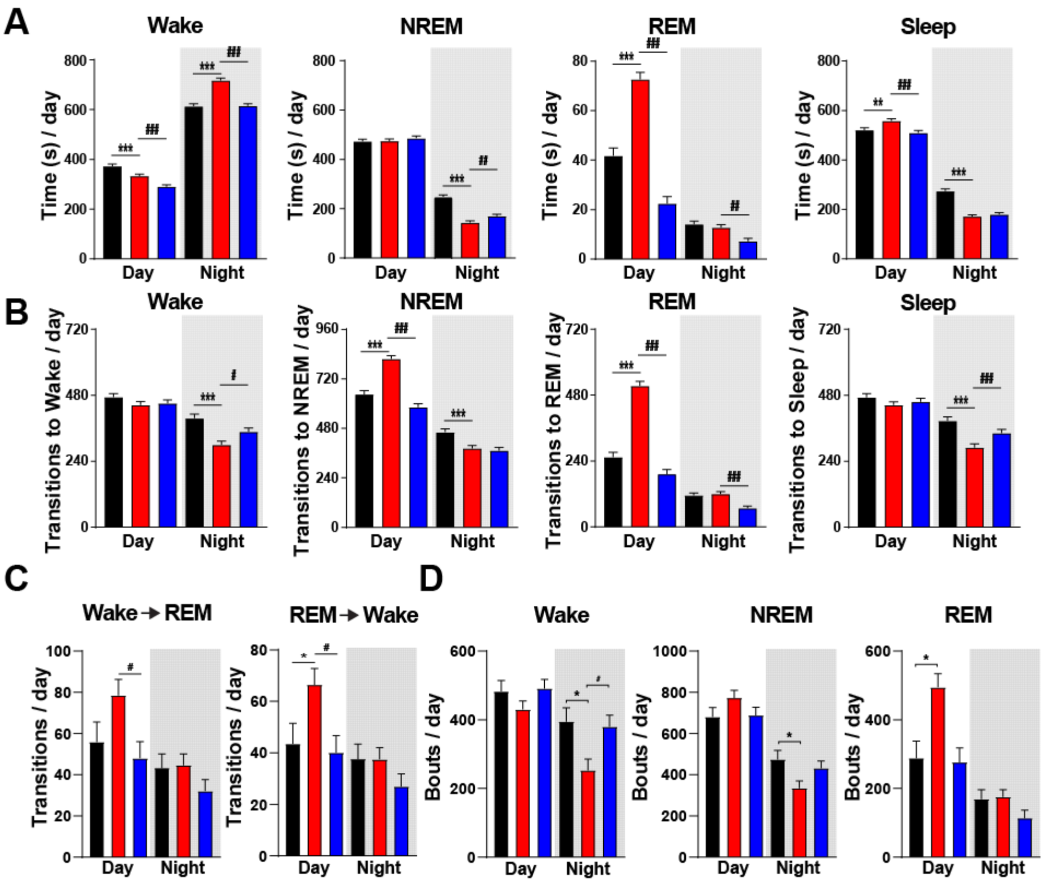

Figure S2. Related to Figure 3. 5xFAD-dependent sleep alterations are rescued by blocking fibrin–microglia interactions in 5xFAD:*Fgg*$^{\gamma 390–396A}$ mice.

(**A**) Total circadian time per average 7-day 15 min bin in Wake, NREM, REM, and Sleep (NREM & REM).

(**B**) Total average transitions to Wake, NREM, REM, and Sleep per seven-day average.

(**C**) Specific transitions from Wake to REM, and REM to Wake during day and night.

(**D**) Average total number of bouts in Wake, NREM, and REM in the day and night.

Values are mean ± SEM; P values by Generalized Linear Mixed Model (GLMM) accounting for repeated measures, individual differences (random factor), fixed factors (day/night and activity levels), and Bonferroni post hoc test for multiple comparisons. *$p < 0.05$, **$p < 0.01$, and ***$p < 0.001$ for Controls vs. 5xFAD; $^{\#}p < 0.05$, $^{\#\#}p < 0.01$, and $^{\#\#\#}p < 0.001$ for 5xFAD vs. 5xFAD:*Fgg*$^{\gamma 390–396A}$.

bioRxiv preprint doi: https://doi.org/10.64898/2026.05.01.722324; this version posted May 6, 2026. The copyright holder for this preprint (which was not certified by peer review) is the author/funder, who has granted bioRxiv a license to display the preprint in perpetuity. It is made available under aCC-BY 4.0 International license.

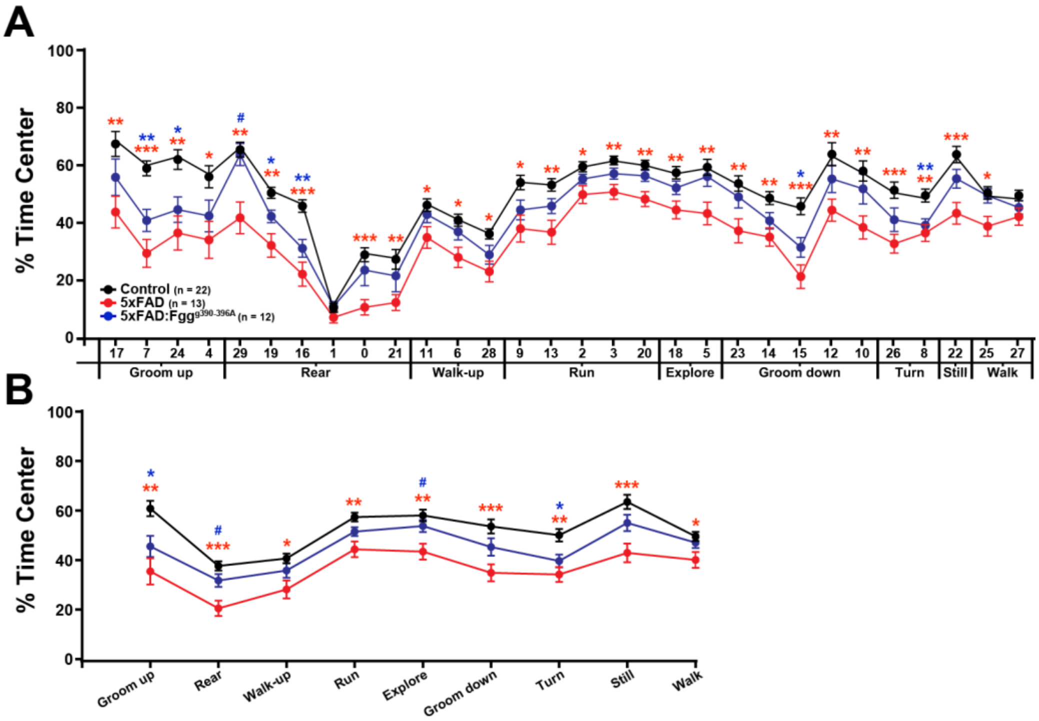

Figure S3. Related to Figure 4. Overall open field spatial analysis shows significantly altered behavior in the center of the arena in 5xFAD mice and recovery across behavioral motifs and communities by blocking fibrin-microglia interactions.

(**A-B**) The overall % of time in the center of the open field for each behavioral motif (A) and across communities (B) in control, 5xFAD, and 5xFAD:*Fgg*$^{\gamma 390–396A}$ mice.

Values are mean ± SEM; P values by repeated two-way ANOVA with Tukey's test for multiple comparisons (A-B); *$p < 0.05$, **$p < 0.01$, and ***$p < 0.001$ (red asterisk) for Controls vs. 5xFAD, (blue asterisk) for Control vs. 5xFAD:*Fgg*$^{\gamma 390–396A}$; $^{\#}p < 0.05$, $^{\#\#}p < 0.01$, and $^{\#\#\#}p < 0.001$ for 5xFAD vs. 5xFAD:*Fgg*$^{\gamma 390–396A}$.

bioRxiv preprint doi: https://doi.org/10.64898/2026.05.01.722324; this version posted May 6, 2026. The copyright holder for this preprint (which was not certified by peer review) is the author/funder, who has granted bioRxiv a license to display the preprint in perpetuity. It is made available under aCC-BY 4.0 International license.

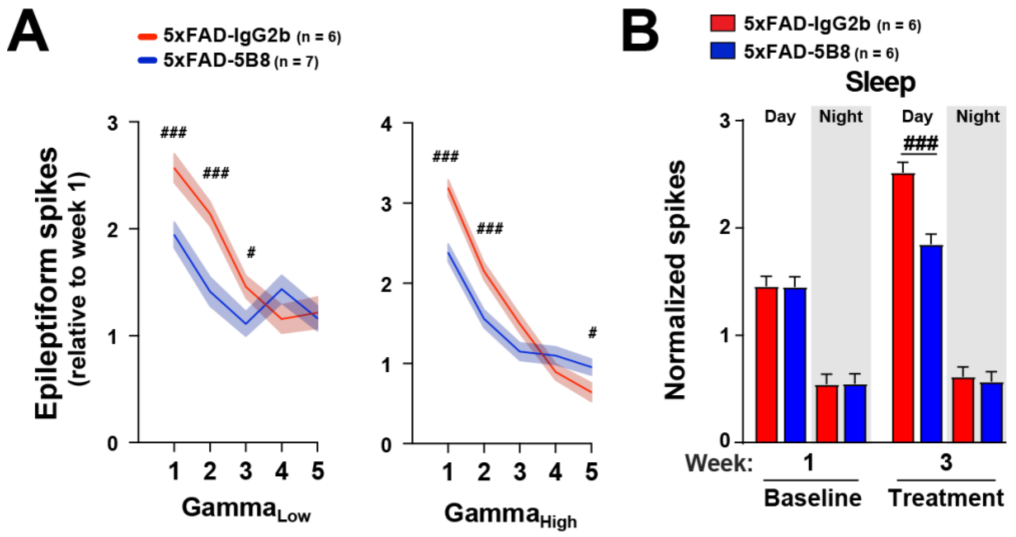

Figure S4. Related to Figure 5. Treatment with the anti-fibrin antibody 5B8 mitigates alterations in Epileptiform spikes observed in low and high gamma, and 5xFAD sleep-dependent alterations.

(A) Epileptic activity per oscillatory power level (low-to-high). 5B8 treatment decreased epileptic activity particularly during periods with low power of both low (30-60 Hz) and high (60-80 Hz) fast-frequency gamma oscillations in 5xFAD mice. (B) Epileptic activity during Sleep in 5B8 treated 5xFAD mice. 5B8 treatment decreased epileptic activity during the day in which the majority of epileptiform spikes occur. Values are mean ± SEM; P values by Univariate General Linear Model (UGLM) accounting for repeated measures, fixed factors (power levels for A; treatment by weeks for B) and Bonferroni post hoc test for multiple comparisons; $^{\#}p < 0.05$, $^{\#\#}p < 0.01$, and $^{\#\#\#}p < 0.001$ for 5xFAD-IgG2b vs. 5xFAD-5B8.
